# Protosappanin B enhances the chemosensitivity of 5-fluorouracil in colon adenocarcinoma by regulating the LINC00612/microRNA-590-3p/Golgi phosphoprotein 3 axis

**DOI:** 10.1007/s12672-024-01036-7

**Published:** 2024-05-28

**Authors:** Zhongshi Hong, Yachen Li, Mingliang Chen, Xiaojing Chen, Xian Deng, Yuze Wu, Chunxiao Wang, Chengzhi Qiu

**Affiliations:** 1 Department of General Surgery, The Second Affiliated Hospital of Fujian Medical University, No.34, Zhongshan North Road, Quanzhou, Fujian 362000 China; 2 Medical Department, The Second Affiliated Hospital of Fujian Medical University, No.34 Zhongshan North Road, Quanzhou, 362000 Fujian China

**Keywords:** Colon adenocarcinoma, Protosappanin B, LINC00612, miR-590-3p, GOLPH3, Chemoresistance, 5-fluorouracil

## Abstract

**Background:**

5-fluorouracil (5-FU) is conventionally used in chemotherapy for colon adenocarcinomas. Acquired resistance of 5-FU remains a clinical challenge in colon cancer, and efforts to develop targeted agents to reduce resistance have not yielded success. Protosappanin B (PSB), the main component of Lignum Sappan extract, is known to exhibit anti-tumor effects. However, whether and how PSB could improve 5-FU resistance in colon cancer have not yet been established. In this study, we aimed to explore the effects and underlying mechanisms of PSB in 5-FU-induced chemoresistance in colon adenocarcinoma.

**Methods:**

Forty-seven paired colon cancer tissue samples from patients who received 5-FU chemotherapy were collected as clinical samples. Two 5-FU resistant colon cancer cell lines were established for in vitro experiments. Reverse transcription-quantitative PCR (RT-qPCR) was performed to determine the mRNA and microRNA (miRNA) expression levels in colon adenocarcinoma tissues and cell lines. Cell Counting Kit-8 (CCK-8) and flow cytometry assays were performed to evaluate cell proliferation and apoptosis, respectively.

**Results:**

LINC00612 was highly expressed in colon adenocarcinoma samples and 5-FU resistant colon cancer cells. LINC00612 knockdown enhances 5-FU chemosensitivity in 5-FU resistant cells. Notably, PSB treatment attenuated LINC00612 expression in 5-FU resistant colon adenocarcinoma cells. Moreover, PSB treatment reversed the increase in LINC00612-induced 5-FU resistance. Mechanistically, LINC00612 specifically bound to miR-590-3p, which promoted 5-FU resistance in colon adenocarcinoma cells and attenuated the inhibitory effect of LINC00612 on GOLPH3 expression.

**Conclusion:**

PSB attenuates 5-FU chemoresistance in colon adenocarcinoma by regulating the LINC00612/miRNA-590-3p/GOLPH3 axis.

**Graphical Abstract:**

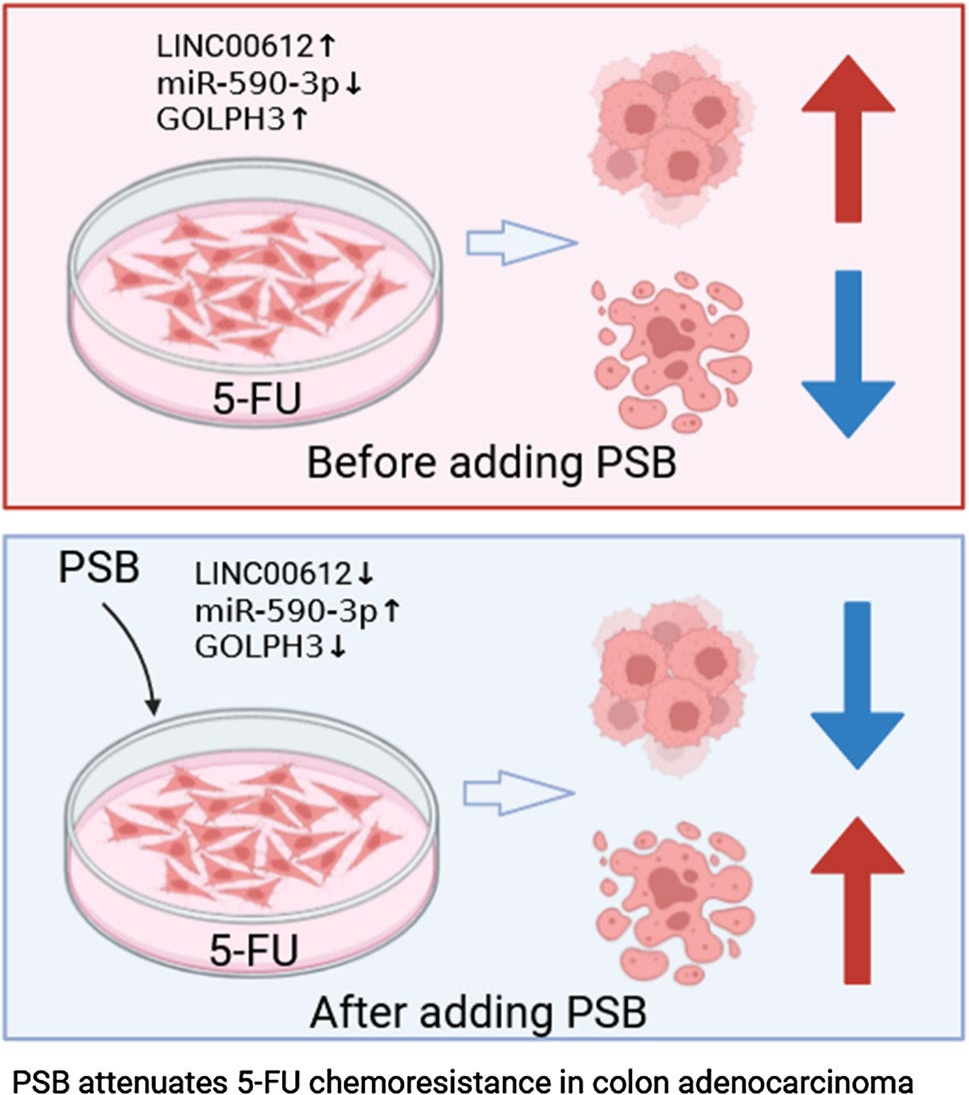

## Highlights

1. LINC00612 is highly expressed in 5-FU resistant colon cancer cells and LINC00612 knockdown enhances 5-FU chemosensitivity in 5-FU resistant cells.
2. PSB reverses the elevated LINC00612-induced 5-FU resistance.
3. PSB attenuates 5-FU chemoresistance in colon adenocarcinoma by regulating the LINC00612/miRNA-590-3p/GOLPH3 axis.

## Introduction

The National Comprehensive Cancer Network (NCCN) guidelines recommend oxaliplatin, fluorouracil, 5-fluorouracil (5-FU), capecitabine, FOLFOX (the combinational strategy of folinic acid, 5-FU, and oxaliplatin), and other chemotherapy regimens for treating colon cancer [1], all of which comprise 5-FU or 5-FU derivatives. Therefore, 5-FU-based comprehensive chemotherapy regimens are considered the gold standard for treating intermediate and advanced colon cancer [2]. However, with the widespread development of chemotherapy, 5-FU resistance has become increasingly prominent, seriously impacting the prognosis of patients and is the main cause of chemotherapy failure in patients [3, 4]. Hence, enhancing and reestablishing the sensitivity of tumor cells to chemotherapeutic drugs and improving clinical efficacy could be valuable in anti-tumor therapy.

Long non-coding RNA (lncRNA) is a sub-type of RNA elements that lack protein-coding capability [5, 6]. LncRNAs promote chemotherapeutic resistance through various pathways, such as increasing drug metabolism, enhancing drug efflux, altering the cell cycle, abnormal apoptosis, and epithelial-mesenchymal transformation [7]. For instance, lncRNA KCNQ1OT1 can inhibit the chemosensitivity of oxaliplatin in colon cancer [8]. Exosomal lncRNA H19 secreted by carcinoma-associated fibroblasts facilitates the chemoresistance of colon adenocarcinoma [9]. Considering 5-FU chemoresistance, lncRNA HCG11 and lncRNA FGD5 were found to modulate 5-FU resistant colon cancer cells by acting as competitive endogenous RNAs to sponge microRNAs (miRNAs) and further regulate target genes [10, 11]. LINC00612 is a newly discovered lncRNA, and was fistly reported in bladder cancer in 2019 [12]. Furhermore, LINC00612 also accelerate tumor progress of osteosarcoma [13]. However, whether LINC00612 modulates chemoresistance in tumor remains unclear.

Traditional Chinese medicines based on natural products have long been employed in combination with conventional treatments to treat patients with various types of cancer [14]. A treatment regimen comprising Poria, Hedyotis diffusa, Scutellaria barbata, Curcuma, Atractylodes, Astragalus, Coix Seed, Codonopsis combined with 5-FU reportedly improved the therapeutic potential of 5-FU-based chemotherapy regimens in colorectal cancer [15]. Growing evidence has demonstrated that natural products can modulate lncRNAs to participate in the regulation of chemoresistance of tumors [16–18]. Protosappanin B (PSB), a *Caesalpinia sappan* Linn extract, has been investigated and could suppress various tumor processes [19–21]. However, whether PSB alleviates chemoresistance of colon adenocarcinoma remains unclear.

In the current study, we aimed to select a candidate lncRNA from newly discovered tumor lncRNAs and examine the functions and molecular mechanisms of 5-FU-resistance in colon adenocarcinoma in vitro and in vivo. Briefly, LINC00612 expression levels in colon adenocarcinoma cell lines were examined, and the effects of LINC00612 knockdown on the proliferation and apoptosis of colon adenocarcinoma 5-FU-resistant cells were analyzed. Moreover, the nude mouse model of subcutaneous tumorigenesis was established to investigate the relationship between PSB and sensitivity to chemotherapy. The role of the LINC00612/miR-590-3p/Golgi phosphoprotein 3 (GOLPH3) axis was determined in 5-FU-resistance colon adenocarcinoma cells.

## Materials and methods

### Bioinformatic analyses

Online databases Starbase 3.0 (http://starbase.sysu.edu.cn/) and Targetscan 7.1 (http://www.targetscan.org/vert_71/) were used to predict interactions between mRNA and miRNA [22–24].

### Specimen collection

Forty-seven pairs of colon adenocarcinoma tissues and matched noncancerous samples (3–5 cm from the distal part of the tumor) were acquired from patients clinically diagnosed at The Second Affiliated Hospital of Fujian Medical University. All included patients underwent three cycles of 5-FU (425 mg/m^2^ administered intravenously for 6–8 h for 5 days and 4 weeks as a treatment cycle) and were assessed for disease progression using the Response Evaluation Criteria in Solid Tumors version 1.1 (RECIST 1.1) [25]. Written informed consent was obtained from all participants. The methodologies employed in the present study were in accordance with the criteria established by the Ethics Committee of The Second Affiliated Hospital of Fujian Medical University (approval no. 2021336, Quanzhou, Fujian, China).

### Candidate lncRNA selection

Through a literature search of the PubMed website, we selected five lncRNAs with the latest studies on the regulation of tumors as research objects for screening. GAS5 is associated with chemoresistance in leukemia, cervical, breast, ovarian, prostate, urinary bladder, lung, gastric, colorectal, liver, osteosarcoma, and brain cancers [26–30]. LINC00294 [31–33], LINC00612 [12, 13], LINC01197 [34–36] and PWAR6 [37–39] are four newly discovered lncRNAs that have been reported to modulate tumor progress.

### Establishment of 5-FU-resistant cells

Colon adenocarcinoma SW620 and LOVO cells and normal colonic mucosal epithelial cells NCM460 were cultured in Dulbecco’s modified Eagle’s medium (DMEM)/F12 medium supplemented with 10% inactivated fetal bovine serum (Gibco, USA), which was replaced every 2 to 3 days. Cells exhibiting 80–90% confluency were digested and used in subsequent experiments. SW620 and LOVO resistant cells (5-FU SW620 and 5-FU LOVO) were established using gradually increasing 5-FU concentrations from 2 to 40 μM. [11]

### Cell treatment

To verify the successful establishment of resistant cells, SW620 and LOVO cells were treated with 5-FU (0, 4, 8, 16, 32, and 64 μM; Nanjing Chia-Tai Tianqing Pharmaceutical Company) along with resistant cells to establish cell viability [19]. Appropriate concentrations of PSB were screened for subsequent experiments by treating 5-FU SW620 and 5-FU LOVO cells with different concentrations of PSB (Shanghai Yuanye Bio-Technology Company) to measure cell viability.

### Lentiviral transfection and RNA interference [40]

Briefly, logarithmic growth 5-FU SW620 and 5-FU LOVO cells were digested and cultured in 6-well plates overnight. All transfection plasmids, including small RNA interfering against LINC00612 (si-LINC00612 1# and si-LINC00612 2#), small RNA interference negative control (si-NC), elevated LINC00612 (LINC00612), blank overexpression vector (Vector), miR-590-3p mimic, mimic nc, si-GOLPH3 1#, si-GOLPH3 2#, short hairpin RNA of LINC00612 (sh-LINC00612), and sh-NC, were purchased from Shanghai GenePharma Company (China). Lipofectamine 3000 (Invitrogen, USA) was used to transfect the corresponding plasmids into the cells according to the manufacturer’s instructions. Overexpression plasmid transfection: Serum-free medium (250 μL) was used to dilute the transfection reagent Lipofectamine 3000 (12.5 μL). The overexpression plasmid (4.2 μg) and P3000 (8 μL) were added to serum-free culture medium (250 μL) and mixed and incubated at room temperature for 5 min. Finally, the solution was mixed and incubated at room temperature for 15 min. Transfection of siRNA and miR: The serum-free medium (250 μL) was used to dilute the transfection reagent Lipofectamine 3000 (12.5 μL) (solution A). The siRNA or miR (12.5 μL) was added to serum-free culture medium (250 μL) to adjust the final concentration of siRNA or miR to 50 nM. The samples were then mixed and incubated at room temperature for 5 min. The liquids were then evenly mixed and incubated at room temperature for 15 min.

### Reverse transcription-quantitative PCR (RT-qPCR) [41]

Total RNA was extracted from colon adenocarcinoma clinical samples and cells using an RNA extraction kit (Beijing Tiengen Biochemical, China). cDNA was obtained using a reverse transcription kit (RIBOBIO, China). The PCR reaction was conducted according to the SYBR Premix Ex Taq kit instructions (Takara, Japan), with pre-denaturation at 95 °C for 30 s, followed by 40 cycles of denaturation at 95 °C for 5 s, and annealing at 60 °C for 30 s. GAPDH was used as a housekeeping control for LINC00612 and GOLPH3. U6 was used as the housekeeping control for miR-590-3p. Data were analyzed using the 2−ΔΔCt relative expression method [8]. All experiments were performed in triplicates. All primer sequences are listed in Table 1.

**Table 1:** Primer sequences

Primer	Forward (5′ → 3′)	Reverse (5′ → 3′)
GAS5	TATGGTGCTGGGTGCGGAT	CCAATGGCTTGAGTTAGGCTT
LINC00294	TGTGTTGTCCTCCAGAATCG	CCAACCAAGAGCCAACAAAG
LINC00612	GGCAGAGCCATGTGTTGGATA	GTGCTCCCTAATGGCTCACA
LINC01197	CCAAATCCTCGGTGCTGTGA	TGCCTCTGTACGCAGATTCC
PWAR6	CTGTGCCGTTTGGCATAAGA	TCACCACCTCACAGATCACC
GOLPH3	ACATCCCCTCACCAATAACAAC	TAGCCAAATCATACTGCTCGTC
miR-590-3p	AAGGAGCUUACAAUCUAGCUGGG	CAGCUAGAUUGUAAGCUCCUUUU
GAPDH	TGAACGGGAAGCTCACTGG	TCCACCACCCTGTTGCTGTA
U6	CTCGCTTCGGCAGCACATATACT	ACGCTTCACGAATTTGCGTGTC
si-LINC00612 1#	AUCUUUCAAGCUAUUUCACAA	GUGAAAUAGCUUGAAAGAUCU
si-LINC00612 2#	CACCGGTAGATGACAGATTAGATACCGAAGTATCTAATCTGTCATCTACC	AAAAGGTAGATGACAGATTAGATACTTCGGTATCTAATCTGTCATCTACC
si-NC	UUCUAAGAAGCGGUCACGUTT	ACGACACAUAGUCGGUUAATT
sh-LINC00612	CACCGGTAGATGACAGATTAGATACCGAAGTATCTAATCTGTCATCTACC	AAAAGGTAGATGACAGATTAGATACTTCGGTATCTAATCTGTCATCTACC
sh-NC	GATCCGGGAGTTCTCCGAAGGTGTCACGCTCGAGCGTGACACCTTCGGAGAACTCTTTTTG	AATTCAAAAAGAGTTCTCCGAAGGTGTCACGCTCGAGCGTGACACCTTCGGAGAACTC
si-GOLPH3 1#	CAAGGACCGCGAGGGTTAC	TTACGTCTCATTCCACAAGCC
si-GOLPH3 2#	GCCTCCAGAAACGGTCCAG	GTCAATACACCCTTTTCCACCA

### Cell counting kit-8 (CCK-8)

Cells in the logarithmic growth phase were collected and inoculated evenly into 96-well plates (3500 cells/well). Then, 100 μL of DMEM containing 10 μL of CCK-8 was added to each well. Two hours later, absorbance was measured at 450 nm using an enzyme-linked immunoassay. Cell viability was calculated using the CCK-8 Cell Counting Kit (Vazyme Biotech Co., Ltd, China) instructions [13].

### Flow cytometry

The culture medium in the 6-well plate was collected in a flow tube and trypsin was added to each well for digestion. After discarding trypsin, 1 mL of complete culture medium was added to terminate the digestion, and the cells were resuspended and collected in a flow tube. After centrifugation, the supernatant was discarded and 300 μL of binding buffer was added to resuspend the cells according to the instructions of the Cell Apoptosis Double Staining Detection Kit (Nanjing Fcmacs Biotech Company). Subsequently, 4 μL of propidium iodide and Annexin V dye were added for 15 min in the dark. The rate of apoptosis was analyzed using flow cytometry [13].

### Dual-luciferase reporter assay [42]

The miR-590-3p overexpression plasmids and respective control plasmids were co-transfected into 5-FU SW620 and 5-FU LOVO cells with LINC00612 or GOLPH3 3′UTR wild-type (PGL3-LINC00612 3′UTR-WT or PGL3-PTTG1 3′UTR-WT) reporting and mutant reporting vectors (PGL3-LINC00612 3′UTR-MUT or PGL3-PTTG1 3′UTR-MUT), respectively [13]. After 48 h, the medium supernatant was absorbed, and PLB lysate (K801-200; BioVision Tech, USA) was added to each well and lysed for 15 min. The lysate was collected, and luciferase activity was evaluated using a luciferase activity kit.

### Subcutaneous tumorigenesis in nude mice

4-week-old male BALB/c nude mice were purchased, 5 × 10^6^ SW620 cells were inoculated subcutaneously into the right axilla of nude mice with a sterile insulin needle, and tumor formation was observed regularly. Nude mice were randomly divided into five groups: sh-NC, sh-LINC00612, sh-NC + 5-FU, sh-LINC00612 + 5-FU, sh-LINC00612 + 5-FU + PSB. From day 5, 2 nmol sh-nc/sh-LINC00612 was injected intratumoral every 3 days for 8 cycles. Intraperitoneal injection of 30 mg/kg 5-FU [20] was performed every 3 days from the 6th day, for 8 cycles. From day 7 onwards, 20 mg/kg PSB [19] was injected intraperitoneally every three days for eight cycles. Graft size was measured and recorded with Vernier calipers every other week. Graft volume was calculated using the formula: 1/2 × (length × width^2^). After 28 days of feeding, the nude mice were sacrificed by cervical dislocation and the tumors were removed and weighed.

### Statistical analysis

Data analysis was performed using GraphPad Prism (v8.3, GraphPad Software, Inc., La Jolla, CA, USA). Each independent experiment was conducted in triplicates. Data are expressed as mean ± standard deviation. The Student’s t-test was used to compare two groups, and one-way ANOVA was used to compare multiple groups. Statistical significance was set at a p < 0.05.

## Results

### LINC00612 is related to 5-FU resistance in colon adenocarcinoma

**Fig. 1:**
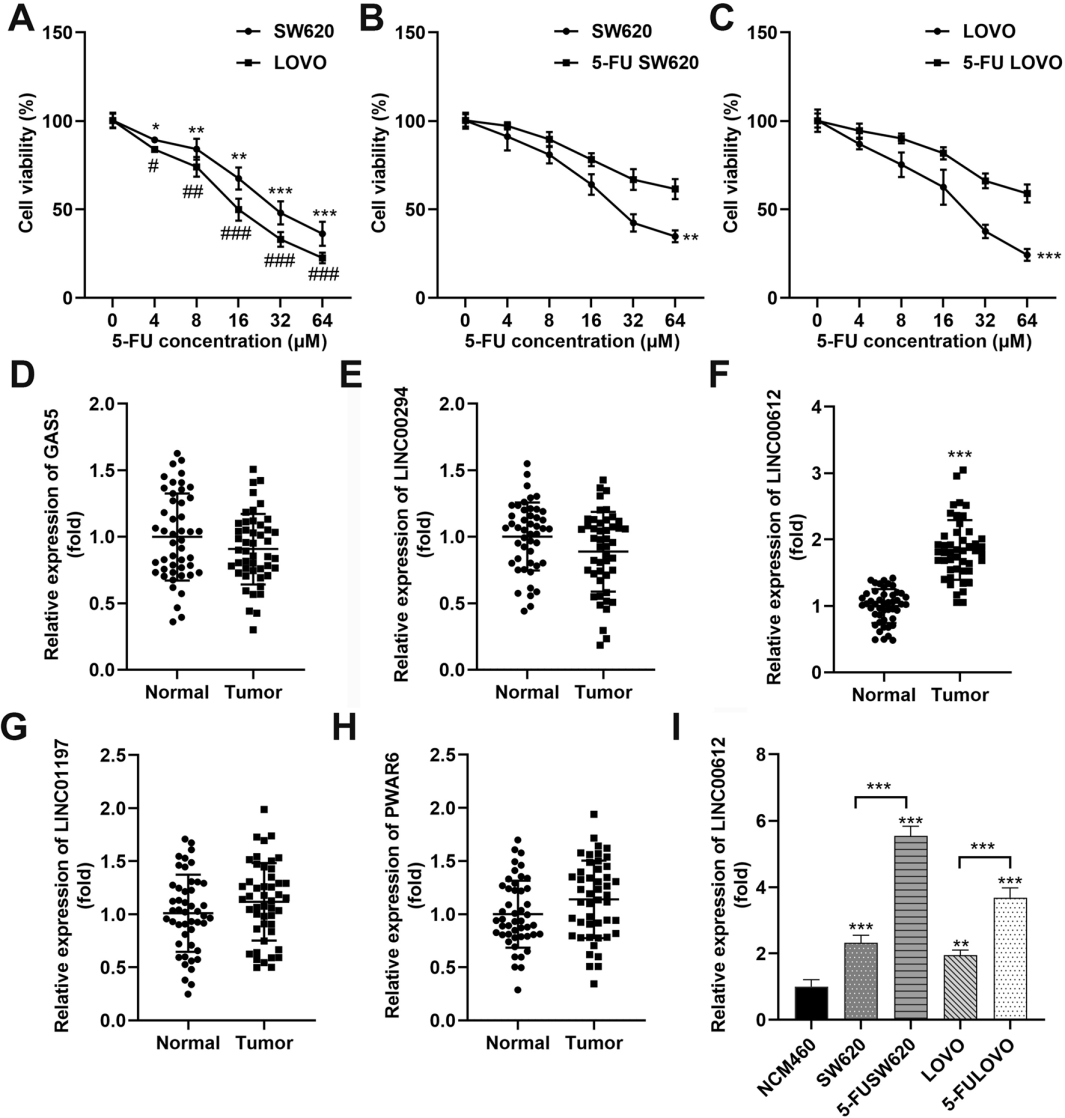
Upregulation of LINC00612 is related to 5-FU resistance in colon adenocarcinoma. 5-FU cytotoxicity for **A** SW620 and LOVO (*p < 0.05, **p < 0.01, ***p < 0.001, compared with SW60-0 μM. #*p* < 0.05, ##*p* < 0.01, ###*p* < 0.001, compared with LOVO-0 μM), **B** 5-FU SW620 (**p < 0.01, compared with SW60), and **C** 5-FU LOVO (***p < 0.001, compared with LOVO) cells were assessed using the cell viability assay. **D** lncRNA GAS5, **E** LINC00294, **F** LINC00612 (***p < 0.01, compared with normal), **G** LNC01197, and **H** lncRNA PWAR6 expression levels in colon adenocarcinoma tissues and normal controls were evaluated using RT-qPCR. **I** LINC00612 expression in NCM460, SW620, and LOVO cells (***p < 0.001 compared with SW60 cells. ###*p* < 0.001 compared with LOVO). 5-FU: 5-fluorouracil; PSB: Protosappanin B; RT-qPCR: reverse transcription quantitative PCR

We first examined 5-FU-induced cytotoxicity in the SW620 and LOVO cell lines using the CCK-8 assay. The half-maximal inhibitory concentrations (IC50) values were 32 and 16 μM in SW620 and LOVO cells, respectively (Fig. 1A). Additionally, 5-FU resistance was characterized in 5-FU SW620 and 5-FU LOVO cells. Following treatment with increasing 5-FU doses, the IC50 values of SW620 and LOVO cells were less than 32 μM, whereas those of 5-FU SW620 and 5-FU LOVO cells were approximately 64 μM, which was approximately twofold higher than that of the parent cell line (Fig. 1B, C). Next, we selected and explored several newly discovered lncRNAs in tumors and detected their expression levels in clinical samples. The expression level of LINC00612 was significantly upregulated in colon adenocarcinoma tissues (Fig. 1D–H). Furthermore, LINC00612 expression was aberrantly elevated in colon cancer cells compared to that in NCM460 cells. Meanwhile, LINC00612 expression was further elevated in 5-FU resistant SW620 and 5-FU resistant LOVO cells compared with the untreated SW620 and LOVO cells (Fig. 1I).

### LINC00612 knockdown promotes sensitivity of 5-FU chemotherapy in 5-FU SW620 and 5-FU LOVO cells

**Fig. 2:**
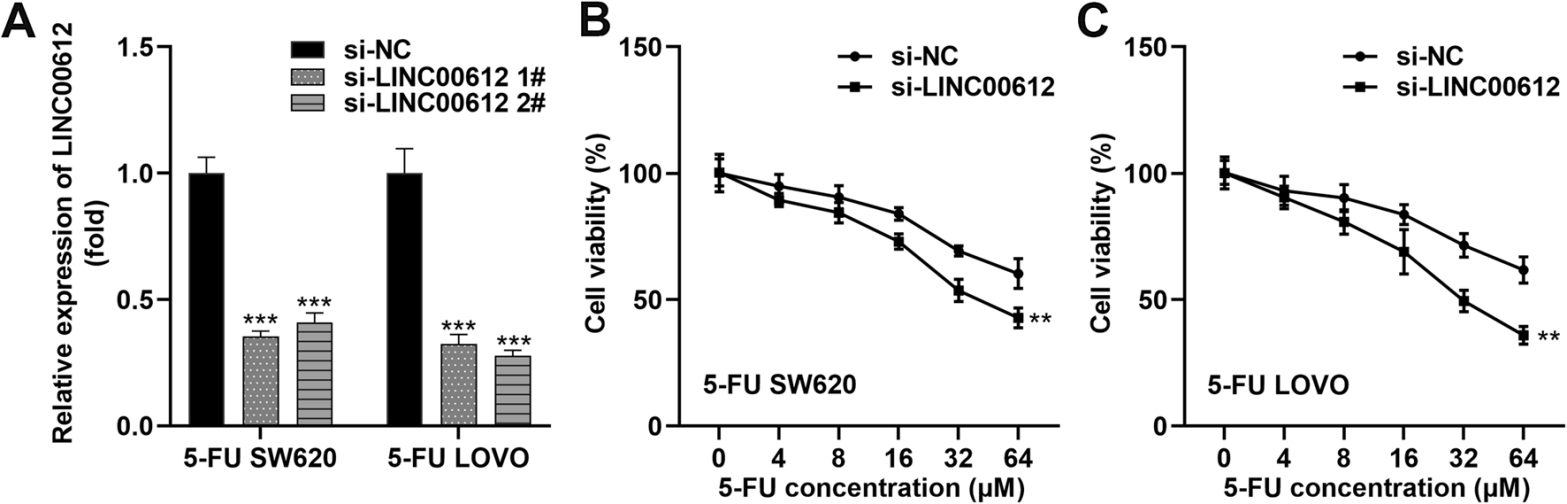
LINC00612 knockdown promotes sensitivity of 5-FU chemotherapy in 5-FU SW620 and 5-FU LOVO cells. **A** Transfection efficiency of LINC00612 silencing in 5-FU SW620 and 5-FU LOVO cells as determined by RT-qPCR assay (***p < 0.001, compared with si-NC). IC50 values of **B** 5-FU SW620 (**p < 0.01, compared with si-NC) and **C** 5-FU LOVO (**p < 0.01, compared with si-NC) cells under the treatment of 5-FU were assessed using the cell viability assay. 5-FU: 5-fluorouracil; PSB: Protosappanin B; RT-qPCR: reverse transcription-quantitative PCR

To examine the effect of LINC00612 on 5-FU induced chemoresistance in 5-FU SW620 and 5-FU LOVO cells, we transfected cells with si-LINC00612 to silence LINC00612 expression (Fig. 2A). In 5-FU SW620 and 5-FU LOVO cells, we observed that cell viability was reduced by about half after 32 μM of 5-FU treatment in the si-LINC00612 group. Meanwhile, inhibition of LINC00612 markedly suppressed cell viability compared with the si-NC group (Fig. 2B, C).

### PSB treatment attenuates colon cancer cell growth by suppressing LINC00612 expression

**Fig. 3:**
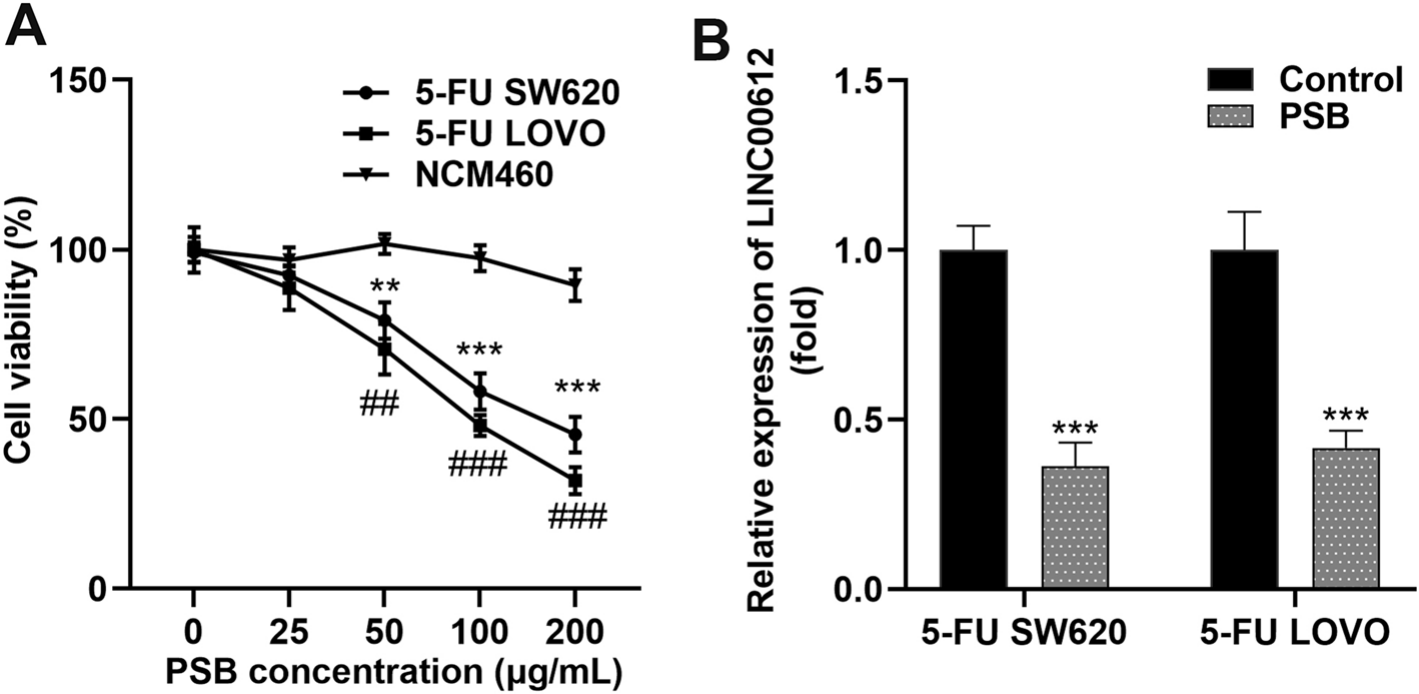
PSB treatment suppresses cell viability and downregulates LINC00612 expression of 5-FU SW620 and 5-FU LOVO cells. **A** Cell viability of 5-FU SW620 and 5-FU LOVO cells determined by CCK-8 assay under the treatment of PSB (***p < 0.001, compared with 5-FU SW620, 0 μg/mL. ##*p* < 0.01, ###*p* < 0.001, compared with 5-FU LOVO, 0 μg/mL). **B** LINC00612 expression in 5-FU SW620 and 5-FU LOVO cells under the treatment of PSB were assessed using RT-qPCR assay. CCK-8, Cell Counting Kit-8; 5-FU: 5-fluorouracil; PSB: Protosappanin B; RT-qPCR: reverse transcription-quantitative PCR

**Fig. 4:**
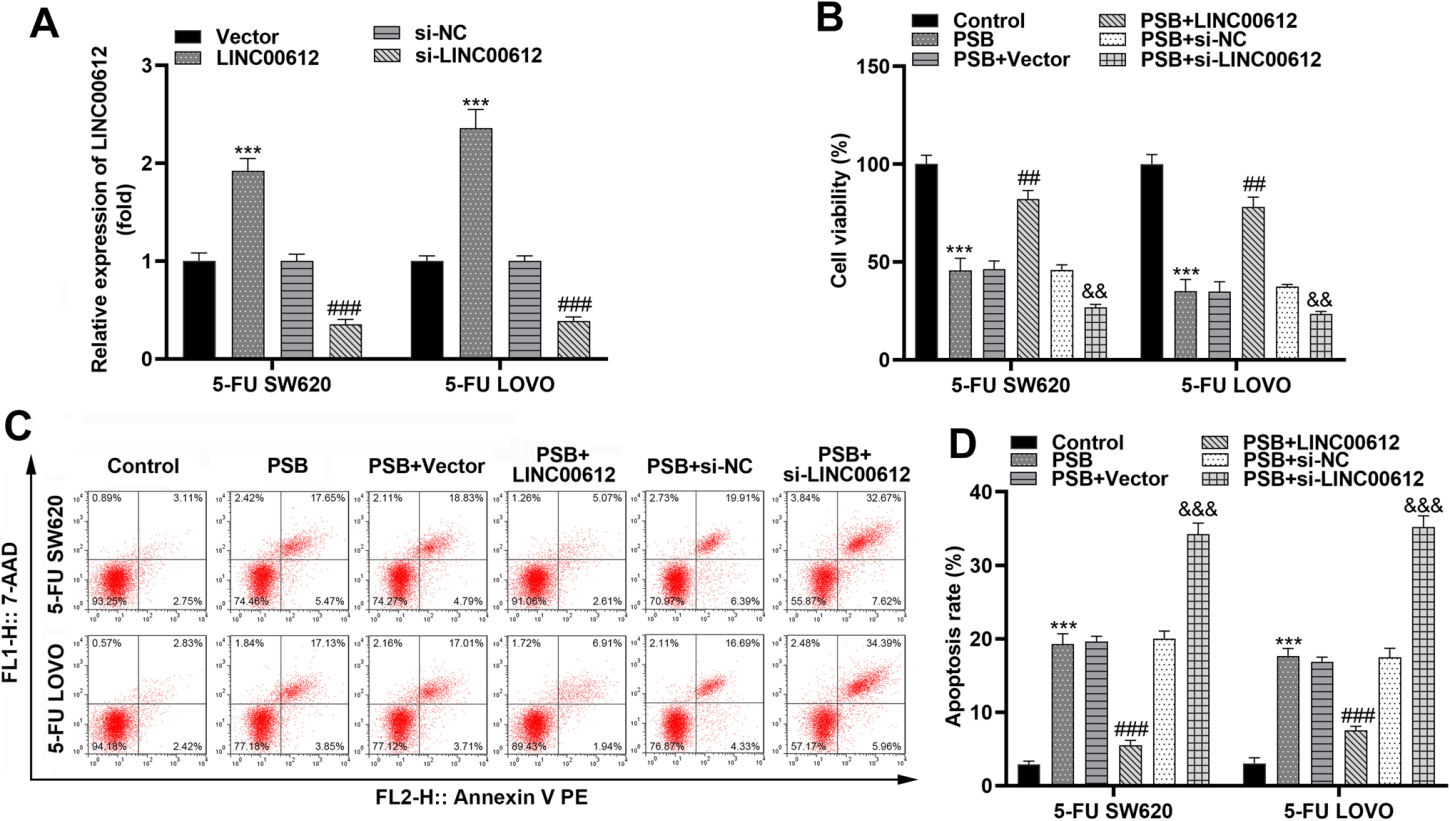
Overexpression of LINC00612 suppresses the effect of PSB on colon cancer cell growth. **A** Transfection efficiency of LINC00612 overexpression and knockdown in 5-FU SW620 and 5-FU LOVO cells determined by RT-qPCR assay (**p < 0.01, ***p < 0.001, compared with Vector. ###*p* < 0.001, compared with si-NC). **B** Cell viability of 5-FU SW620 and 5-FU LOVO cells measured by CCK-8 assay (***p < 0.001, compared with control. ##*p* < 0.01, compared with PSB + Vector. &&*p* < 0.01, compared with PSB + si-NC). **C** Flow cytometry scatter plots of 5-FU SW620 and 5-FU LOVO cells. **D** Apoptosis rate of 5-FU SW620 and 5-FU LOVO cells (***p < 0.001, compared with control. ###*p* < 0.01, compared with PSB + Vector. &&&*p* < 0.001, compared with PSB + si-NC). 5-FU: 5-fluorouracil; PSB: Protosappanin B; CCK-8: Cell Counting Kit-8; RT-qPCR: reverse transcription-quantitative PCR

Subsequently, the NCM460, 5-FU SW620, and 5-FU LOVO cells were treated with PSB. PSB significantly inhibited 5-FU SW620 and 5-FU LOVO cell growth in a dose-dependent manner, and even at a high concentration of 200 μg/mL, PSB was less toxic to normal cells (Fig. 3A). Furthermore, 100 μg/mL PSB, which reduced the number of 5-FU SW620 and 5-FU LOVO cells by 50%, was selected as the optimal concentration. PSB treatment decreased LINC00612 expression, as evaluated by RT-qPCR in 5-FU SW620 and 5-FU LOVO cells (Fig. 3B). To confirm whether the PSB-induced decline in LINC00612 expression could affect the functions of 5-FU SW620 and 5-FU LOVO cells, we established LINC00612 overexpressing cells and LINC00612 knockdown cells to perform a reverse validation experiment (Fig. 4A). Cell viability was assessed by the CCK-8 assay, and apoptosis was evaluated by flow cytometry. PSB markedly suppressed cell viability and facilitated apoptosis, and inhibition of LINC00612 dramatically further aggravated the effects of PSB on colon cancer cells, whereas elevated LINC00612 expression markedly reversed these effects (Fig. 4B–D).

### PSB attenuates cell aggressiveness through the LINC00612/miRNA-590-3p/GOLPH3 axis

**Fig. 5:**
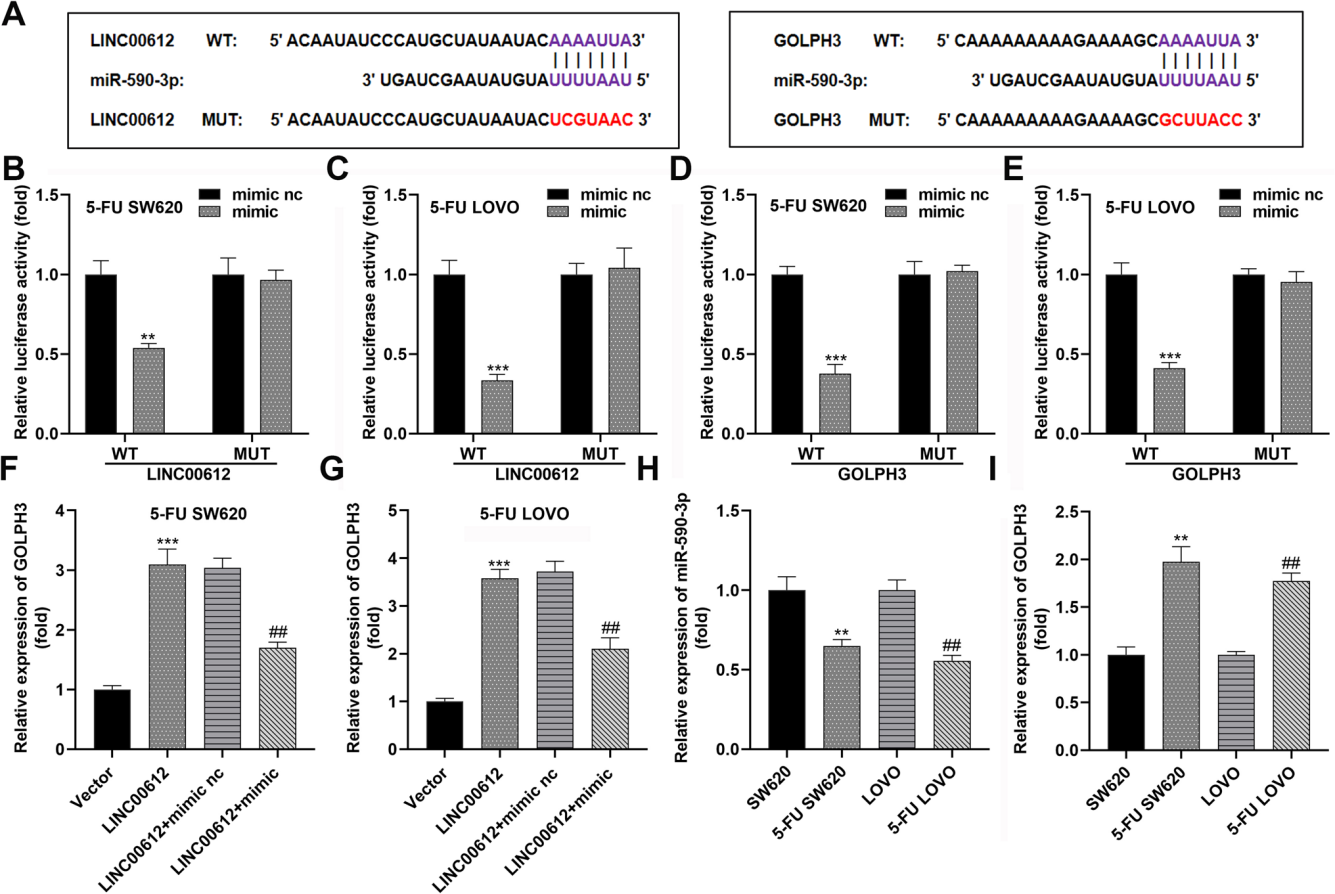
LINC00612 sequesters miR-590-3p away from GOLPH3 in colon adenocarcinoma cells. **A** Prediction of miR-590-3p and its target RNA-binding site oligonucleotides. **B**, **C** Luciferase activity of wildtype-LINC00612 and mutant-LINC00612 in 5-FU SW620 and 5-FU LOVO cells with elevated miR-590-3p (**p < 0.01, ***p < 0.001, compared with mimic NC). **D**, **E** Luciferase activity of wildtype-GOLPH3 and mutant-GOLPH3 in 5-FU SW620 and 5-FU LOVO cells with elevated miR-590-3p (***p < 0.001, compared with mimic NC). **F**, **G** GOLPH3 expression in 5-FU SW620 and 5-FU LOVO cells with elevated LINC00612 and miR-590-3p levels (***p < 0.001, compared with the vector. ##*p* < 0.01, compared to the LINC00612 + mimic NC). **H** miR-590-3p and **I** GOLPH3 expression in SW620 and LOVO cells and chemotherapy-resistant cells (**p < 0.01, compared to SW620 cells. ##*p* < 0.01, compared to LOVO). 5-FU: 5-fluorouracil; PSB: protosappanin B

**Fig. 6:**
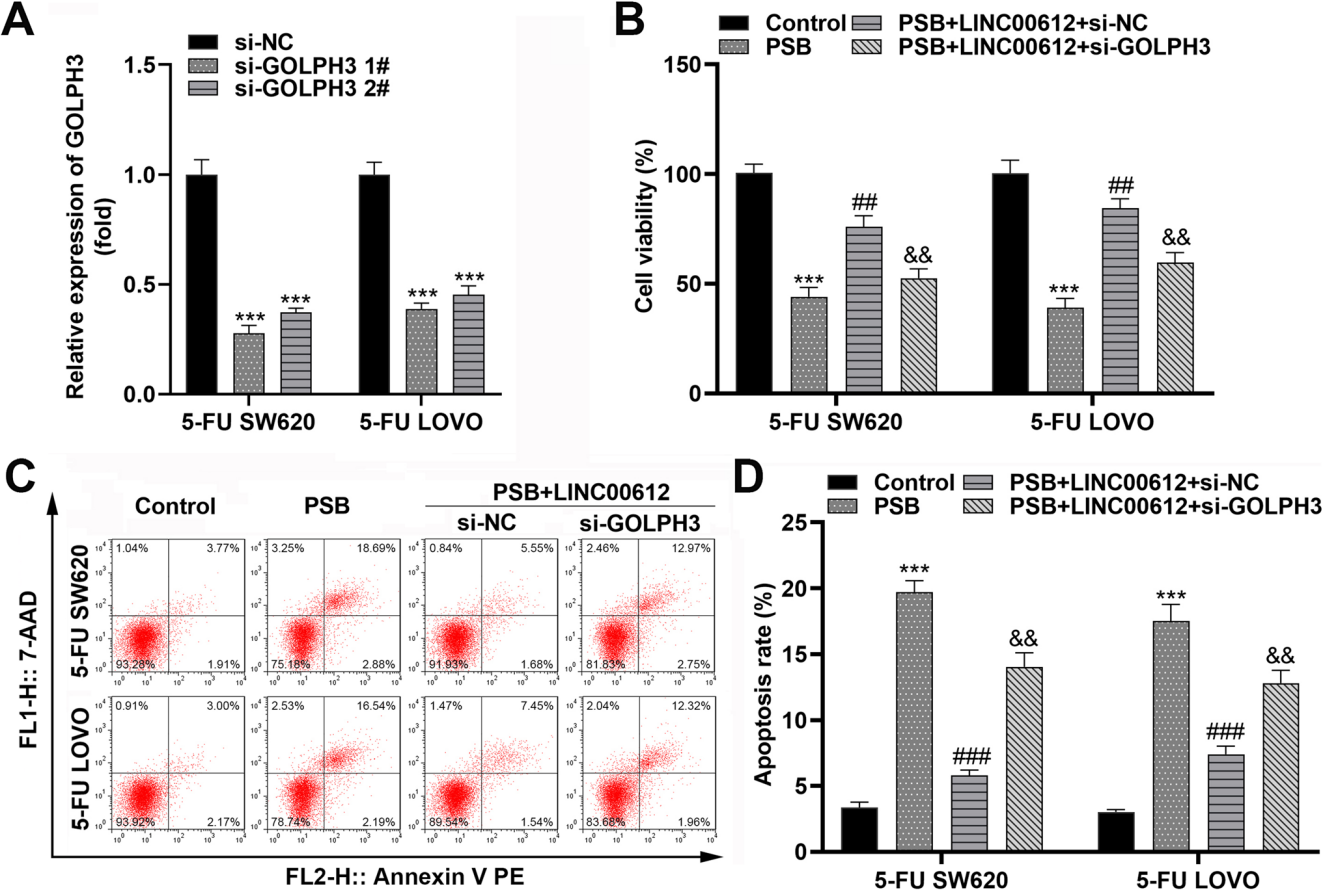
PSB attenuates cell aggressiveness via the LINC00612/miRNA-590-3p/GOLPH3 axis. **A** Transfection efficiency of GOLPH3 silencing in 5-FU SW620 and 5-FU LOVO cells performed by RT-qPCR (***p < 0.001, compared with si-NC). **B** Cell viability of 5-FU SW620 and 5-FU LOVO cells measured by CCK-8 assay (***p < 0.001, compared with control. ##*p* < 0.01, compared with PSB. &&*p* < 0.01, compared with PSB + LINC00612 + si-NC). **C** Flow cytometry scatter plots of 5-FU SW620 and 5-FU LOVO cells. **D** Apoptosis rate of 5-FU SW620 and 5-FU LOVO cells (***p < 0.001, compared with control. ###*p* < 0.01, compared with PSB. &&*p* < 0.01, compared with PSB + LINC00612 + si-NC). 5-FU: 5-fluorouracil; PSB: Protosappanin B; RT-qPCR: reverse transcription-quantitative PCR

Given that lncRNAs adsorb miRNAs to modulate downstream target mRNA, we further explored the LINC00612-mediated molecular mechanism in colon adenocarcinoma progression. First, miR-590-3p was found to bind to LINC00612 and its target gene GOLPH3 (Fig. 5A). Luciferase assays revealed that miR-590-3p overexpression significantly attenuated the luciferase activity of wildtype-LINC00612 and wildtype-GOLPH3, but had no effect on mutant-LINC00612 and mutant-GOLPH3 in 5-FU SW620 and 5-FU LOVO cells (Fig. 5B–E). Furthermore, LINC00612 increased GOLPH3 expression, which was downregulated by miR-590-3p in 5-FU SW620 and 5-FU LOVO cells (Fig. 5F, G). miR-590-3p exhibited lower expression, whereas GOLPH3 showed higher expression in 5-FU resistant cells than in parental cells (Fig. 5H, I). Subsequently, we silenced GOLPH3 in both 5-FU SW620 and 5-FU LOVO cells (Fig. 6A). Elevated LINC00612 expression reversed PSB-induced inhibition of cell viability and PSB-induced cell apoptosis. Furthermore, GOLPH3 inhibition markedly reversed the effects of LINC00612 (Fig. 6B–D).

### PSB promotes chemosensitivity of 5-FU in vivo

**Fig. 7:**
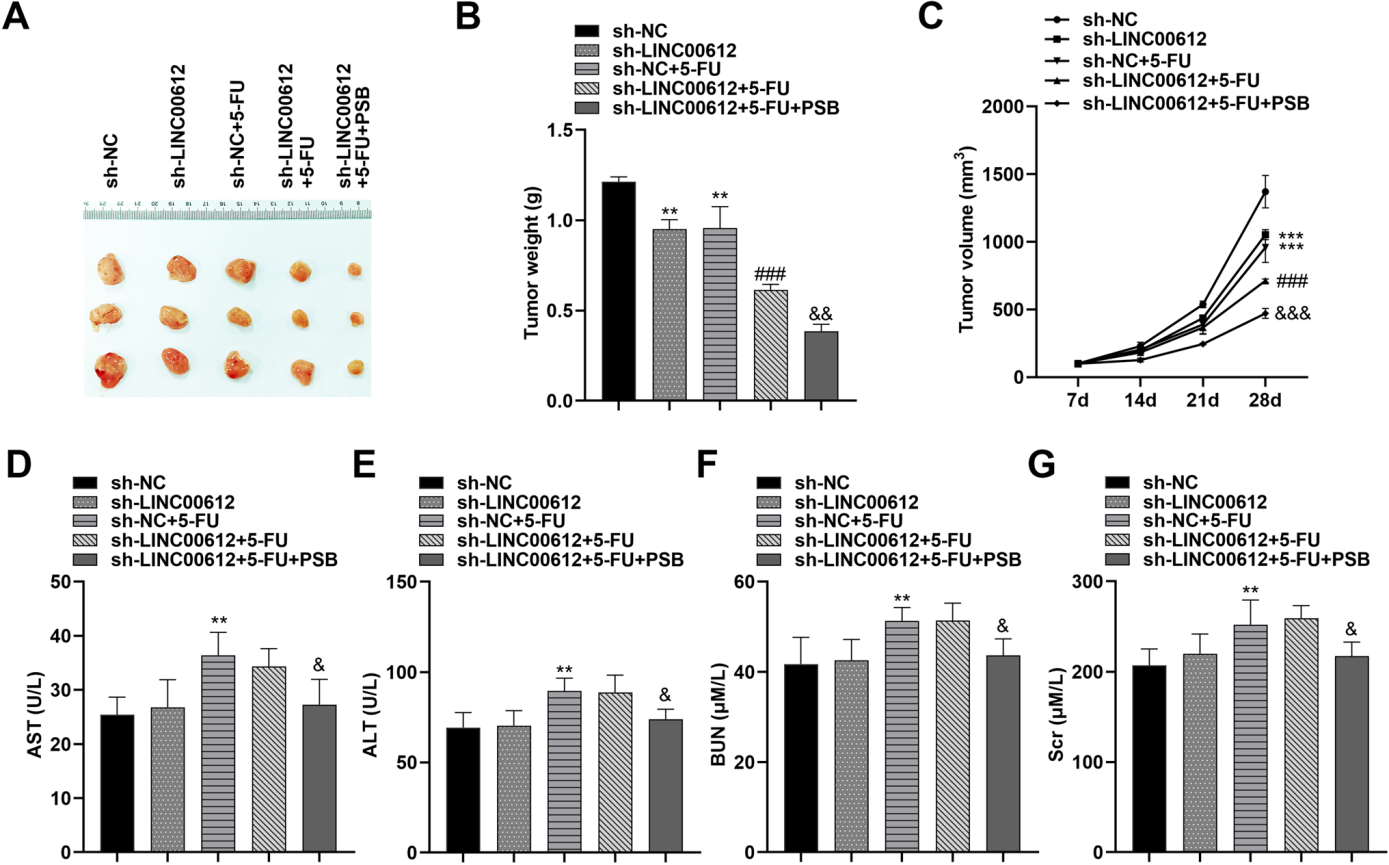
PSB promotes chemosensitivity of 5-FU in vivo. **A** Images of tumors, **B** tumor weight, and **C** tumor volume of nodules from mice treated with 5-FU and PSB. Effects of PSB on **D** ALT, **E** AST), **F** BuN, **G** Scr in mice. **p < 0.01, ***p < 0.001, compared with sh-NC. ###*p* < 0.001 compared to sh-NC + 5-FU. &*p* < 0.05, &&*p* < 0.01, &&&*p* < 0.001, compared with sh-LINC00612 + 5-FU). 5-FU: 5-fluorouracil; PSB: protosappanin B

A nude mouse model of subcutaneous tumorigenesis was established to investigate the relationship between PSB and sensitivity to chemotherapy (Fig. 7A). In the subcutaneous tumorigenesis experiment of SW620 cells, compared with the sh-NC group, the weight (Fig. 7B) and volume (Fig. 7C) of tumor tissue in the sh-LINC00612 group was significantly reduced, which indicated that the malignant process of the tumor was inhibited after inhibiting the expression of LINC00612. The combination of sh-LINC00612 and 5-FU had a stronger anti-tumor effect than sh-LINC00612 or 5-FU alone, and PSB treatment further inhibited tumor growth and enhanced the chemotherapy sensitivity of 5-FU. To evaluate the acute toxicity of PSB in vivo, the activity of alanine transaminase (ALT), aspartate transaminase (AST), serum creatinine (Scr), and blood urea nitrogen (BUN) were measured. The levels of AST, ALT, BUN, and Scr were up-regulated significantly after 5-FU treatment, while the expression in serum with a reversing trend to normal induced by PSB treatment compared with those in the control group (Fig. 7D–G). The results suggested that PSB could alleviate the liver and kidney dysfunction in tumor xenograft mice.

## Discussion

In the present study, PSB suppressed cell viability and accelerated the apoptosis of 5-FU-resistant colon adenocarcinoma cells by regulating the LINC00612/miR-590-3p/GOLPH3 axis (Graphical abstract).

Currently, 5-FU or combination drug therapy remains the first-line treatment for gastrointestinal malignancies; however, the overall effective cure rate is insufficient owing to drug resistance [43]. Types of 5-FU resistance include primary resistance and secondary resistance. Resistance to chemotherapy in colorectal cancer cells is an important factor affecting clinical treatment failure, tumor recurrence, migration, invasion, and disease deterioration [44, 45]. It is reported that when 5-FU is combined with other anti-cancer drugs or 5-FU based regimens (FOLFOX, XELOX, and SOX) the response rates of 5-FU based first-line chemotherapy were raised to 40–50% [43]. Thus, novel therapeutic strategies are of immediate requirement to combat drug resistance and improve drug response rates.

It has been reported that the combination of traditional Chinese medicine monomer and 5-FU can enhance the sensitivity of chemotherapy. Curcumin, Ginkgo biloba exocarp extracts, and Astragaloside IV reverses 5-FU resistance in various tumors [46–48]. Exploring new traditional Chinese medicine monomers to enhance chemotherapy sensitivity is expected to provide new ideas for alleviating chemotherapy resistance in clinic. Additionlly, some lncRNAs are key contributors to various chemoresistance mechanisms and are important determinants of the efficacy of anticancer therapies in cancers. LncRNA HOTAIR contributes to 5-FU resistance through suppressing miR-218 and activating NF-κB/TS signaling in colorectal Cancer [49]. LncRNA FGD5-AS1 promotes glycolysis through modulating the miR-330-3p-HK2 axis, leading to 5-Fu resistance of colorectal cancer cells [50]. Formononetin, an isoflavonoid isolated from astragalus membranaceus and spatholobus suberectus, relieves the chemotherapy resistance through lncRNA AFAP1-AS1-miR-195/miR-545 axis in triple-negative breast cancer [17]. Therefore, the mechanism of Chinese medicine monomer participating in 5-FU resistance by regulating lncRNA is worth studying.

Our data suggest that LINC00612 expression is aberrantly upregulated in 5-FU resistant colon carcinoma cells. Silencing LINC00612 expression downregulated the IC50 value of 5-FU resistant colon carcinoma cells, thereby enhancing their chemosensitivity. PSB, a newly discovered traditional Chinese medicine monomer,has attracted attention for its anti-inflammatory, antibacterial and antioxidant properties [20]. In recent years, its anti-tumor properties have also been discovered. For instance, PSB can inhibit the proliferation of human bladder cancer cell lines and colon cancer cell lines [19]. To be specific, PSB inhibits the proliferation and promotes the apoptosis of human bladder cancer cells via interference with cell cycle regulation [20]. PSB inhibits GOLPH3 expression and intracellular signaling pathways in colon cancer cells [19]. Whether the inhibitory effect of PSB on colon caner is related to drug resistance is unknown. In this study, we found that PSB suppressed the viability of 5-FU resistant cells and inhibited LINC00612 expression. Hence, we speculate that PSB alleviates drug resistance in colon cancer cells by modulating LNC00612, which was substantiated by our experimental findings. Elevated LINC00612 expression reverses the inhibitory effects of PSB on cell growth.

Meanwhile, lncRNAs can serve as miRNA sponges and act as a new type of competing endogenous RNA (ceRNA) regulator to degrade miRNAs [51, 52]. Our data demonstrated that LINC00612 could bind to miR-590-3p. miR-590-3p has been shown to affect the biological function of tumor cells in various cancers, including gastric cancer [53], liver cancer [54], and breast cancer [55]. However, whether miR-590-3p could affect drug resistance in colon cancer warrants further investigation.

GOLPH3, located on chromosome 5p13, is a highly conserved membrane protein in the Golgi complex, which is closely related to structural maintenance, vesicle transport, and Golgi glycosylation [56]. GOLPH3 has been identified as an oncoprotein and is significantly upregulated in prostate cancer [57], esophageal cancer [58], gastric cancer [59], ovarian cancer [60], and colon cancer [61]. Herein, LINC00612 expression positively regulates GOLPH3 expression. Moreover, GOLPH3 knockdown reversed the effects of LINC00612 on 5-FU-resistance in colon adenocarcinoma cells. These results revealed that PSB modulated LINC00612 to sponge miR-590-3p, thereby suppressing GOLPH3 expression in 5-FU-resistant colon adenocarcinoma cells.

However, the limitations of the present study must be addressed. Herein, we examined colon cancer tissues obtained only from Han Chinese patients; hence, the sample capacity should be broadened, and multiple ethnic groups must be investigated.

## Conclusion

The findings of the present study demonstrate the regulation of 5-FU chemoresistance in colon cancer via the LINC00612/miR-590-3p/GOLPH3 pathway. In addition, we confirmed that PSB attenuated 5-FU chemoresistance in colon cancer by targeting LINC00612, which may provide a novel treatment strategy for 5-FU chemoresistance in colon cancer.

## Data Availability

The datasets used and/or analysed during the current study are available from the corresponding author on reasonable request.

## References

[1] Benson AB, Venook AP, Al-Hawary MM, Cederquist L, Chen YJ, Ciombor KK (2018). NCCN guidelines insights: colon cancer, version 2.2018. J Natl Compr Canc Netw.

[2] Guo J, Yu Z, Das M, Huang L (2020). Nano codelivery of oxaliplatin and folinic acid achieves synergistic chemo-immunotherapy with 5-fluorouracil for colorectal cancer and liver metastasis. ACS Nano.

[3] Liang G, Zhu Y, Ali DJ, Tian T, Xu H, Si K (2020). Engineered exosomes for targeted co-delivery of miR-21 inhibitor and chemotherapeutics to reverse drug resistance in colon cancer. J Nanobiotechnol.

[4] Montrose DC, Saha S, Foronda M, Mcnally EM, Chen J, Zhou XK (2021). Exogenous and endogenous sources of serine contribute to colon cancer metabolism, growth, and resistance to 5-fluorouracil. Cancer Res.

[5] Mathieu EL, Belhocine M, Dao LT, Puthier D, Spicuglia S (2014). functions of lncRNA in development and diseases. Med Sci.

[6] Wan J, Liu B (2021). Construction of lncRNA-related ceRNA regulatory network in diabetic subdermal endothelial cells. Bioengineered.

[7] Barth DA, Juracek J, Slaby O, Pichler M, Calin GA (2020). LncRNA and mechanisms of drug resistance in cancers of the genitourinary system. Cancers.

[8] Li Y, Li C, Li D, Yang L, Jin J, Zhang B (2019). LncRNA kcnq1ot1 enhances the chemoresistance of oxaliplatin in colon cancer by targeting the miR-34a/ATG4B pathway. Onco Targets Ther.

[9] Ren J, Ding L, Zhang D, Shi G, Xu Q, Shen S (2018). Carcinoma-associated fibroblasts promote the stemness and chemoresistance of colorectal cancer by transferring exosomal lncRNA H19. Theranostics.

[10] Cui Z, Wang Q, Deng MH, Han QL (2021). LncRNA HCG11 promotes 5-FU resistance of colon cancer cells through reprogramming glucose metabolism by targeting the miR-144–3p-PDK4 axis. Cancer Biomark.

[11] Gao SJ, Ren SN, Liu YT, Yan HW, Chen XB (2021). Targeting EGFR sensitizes 5-Fu-resistant colon cancer cells through modification of the lncRNA-FGD5-AS1-miR-330-3p-Hexokinase 2 axis. Mol Ther Oncol.

[12] Miao L, Liu HY, Zhou C, He X (2019). LINC00612 enhances the proliferation and invasion ability of bladder cancer cells as ceRNA by sponging miR-590 to elevate expression of PHF14. J Exp Clin Cancer Res.

[13] Zhou Y, Li X, Yang H (2020). LINC00612 functions as a ceRNA for miR-214-5p to promote the proliferation and invasion of osteosarcoma in vitro and in vivo. Exp Cell Res.

[14] Dong Y, Chen H, Gao J, Liu Y, Li J, Wang J (2019). Bioactive ingredients in Chinese herbal medicines that target non-coding RNAs: promising new choices for disease treatment. Front Pharmacol.

[15] Chen P, Ni W, Xie T, Sui X (2019). Meta-analysis of 5-fluorouracil-based chemotherapy combined with traditional Chinese medicines for colorectal cancer treatment. Integr Cancer Ther.

[16] Zhou M, Zhang G, Hu J, Zhu Y, Lan H, Shen X (2021). Rutin attenuates sorafenib-induced chemoresistance and autophagy in hepatocellular carcinoma by regulating BANCR/miRNA-590-5P/OLR1 axis. Int J Biol Sci.

[17] Wu J, Xu W, Ma L, Sheng J, Ye M, Chen H (2021). Formononetin relieves the facilitating effect of lncRNA AFAP1-AS1-miR-195/miR-545 axis on progression and chemo-resistance of triple-negative breast cancer. Aging.

[18] Hu T, Fei Z, Su H, Xie R, Chen L (2019). Polydatin inhibits proliferation and promotes apoptosis of doxorubicin-resistant osteosarcoma through lncRNA tug1 mediated suppression of Akt signaling. Toxicol Appl Pharmacol.

[19] Zheng XC, Shi ZS, Qiu CZ, Hong ZS, Wang CX, Zhuang HB (2020). Protosappanin B exerts anti-tumor effects on colon cancer cells via inhibiting GOLPH3 expression. Integr Cancer Ther.

[20] Yang X, Zhao L, Zhang T, Xi J, Liu S, Ren L (2019). Protosappanin B promotes apoptosis and causes G1 cell cycle arrest in human bladder cancer cells. Sci Rep.

[21] Yang X, Ren L, Zhang S, Zhao L, Wang J (2016). Antitumor effects of purified protosappanin B extracted from lignum sappan. Integr Cancer Ther.

[22] Li JH, Liu S, Zhou H, Qu LH, Yang JH (2014). starBase v2.0: decoding miRNA-ceRNA, miRNA-ncRNA and protein-RNA interaction networks from large-scale CLIP-Seq data. Nucleic Acids Res.

[23] Miao L, Yin RX, Zhang QH, Liao PJ, Wang Y, Nie RJ (2019). A novel circRNA-miRNA-mRNA network identifies circ-YOD1 as a biomarker for coronary artery disease. Sci Rep.

[24] Rawat M, Nighot M, Al-Sadi R, Gupta Y, Viszwapriya D, Yochum G (2020). IL1B increases intestinal tight junction permeability by up-regulation of MIR200C-3p, which degrades occludin mRNA. Gastroenterology.

[25] Eisenhauer EA, Therasse P, Bogaerts J, Schwartz LH, Sargent D, Ford R (2009). New response evaluation criteria in solid tumours: revised RECIST guideline (version 1.1). Eur J Cancer.

[26] Lambrou GI, Hatziagapiou K, Zaravinos A (2020). The non-coding RNA GAS5 and its role in tumor therapy-induced resistance. Int J Mol Sci.

[27] Zhou X, Xiao D (2020). Long non-coding RNA GAS5 is critical for maintaining stemness and induces chemoresistance in cancer stem-like cells derived from HCT116. Oncol Lett.

[28] Gao Z, Wang J, Chen D, Ma X, Yang W, Zhe T (2018). Long non-coding RNA GAS5 antagonizes the chemoresistance of pancreatic cancer cells through down-regulation of miR-181c-5p. Biomed Pharmacother.

[29] Fang X, Zhong G, Wang Y, Lin Z, Lin R, Yao T (2020). Low GAS5 expression may predict poor survival and cisplatin resistance in cervical cancer. Cell Death Dis.

[30] Chen Z, Pan T, Jiang D, Jin L, Geng Y, Feng X (2020). The lncRNA-GAS5/miR-221-3p/DKK2 axis modulates ABCB1-mediated adriamycin resistance of breast cancer via the Wnt/β-catenin signaling pathway. Mol Ther Nucleic Acids.

[31] Zhou X, Lv L, Zhang Z, Wei S, Zheng T (2020). LINC00294 negatively modulates cell proliferation in glioma through a neurofilament medium-mediated pathway via interacting with miR-1278. J Gene Med.

[32] Dong X, Pi Q, Yuemaierabola A, Guo W, Tian H (2021). Silencing LINC00294 restores mitochondrial function and inhibits apoptosis of glioma cells under hypoxia via the mir-21-5p/caskin1/camp axis. Oxid Med Cell Longev.

[33] Qiu J, Zhou S, Cheng W, Luo C (2020). LINC00294 induced by GRP78 promotes cervical cancer development by promoting cell cycle transition. Oncol Lett.

[34] Ling J, Wang F, Liu C, Dong X, Xue Y, Jia X (2019). FOXO1-regulated lncRNA LINC01197 inhibits pancreatic adenocarcinoma cell proliferation by restraining Wnt/beta-catenin signaling. J Exp Clin Cancer Res.

[35] Jia YH, Zheng WW, Ye ZH (2020). Clinical significance of CA 19.9 and LINC01197 in pancreatic cancer. Eur Rev Med Pharmacol Sci.

[36] Yue Q, Bai J, Wang F, Xue F, Li L, Duan X (2022). Novel classification and risk model based on ferroptosis-related lncRNAs to predict oncologic outcomes for gastric cancer patients. J Biochem Mol Toxicol.

[37] Huang S, Li Y, Hu J, Li L, Liu Z, Guo H (2021). lncRNA PWAR6 regulates proliferation and migration by epigenetically silencing YAP1 in tumorigenesis of pancreatic ductal adenocarcinoma. J Cell Mol Med.

[38] Zhang C, Liu H, Xu P, Tan Y, Xu Y, Wang L (2021). Identification and validation of a five-lncRNA prognostic signature related to glioma using bioinformatics analysis. BMC Cancer.

[39] Lin X, Jiang T, Bai J, Li J, Wang T, Xiao J (2018). Characterization of transcriptome transition associates long noncoding RNAs with glioma progression. Mol Ther Nucleic Acids.

[40] Yu C, Liang Y, Jin Y, Li Q (2021). lncRNA GAS5 enhances radiosensitivity of hepatocellular carcinoma and restricts tumor growth and metastasis by miR-144-5p/ATF2. Am J Transl Res.

[41] Zha Z, Han Q, Liu W, Huo S (2020). lncRNA GAS8-AS1 downregulates lncRNA UCA1 to inhibit osteosarcoma cell migration and invasion. J Orthop Surg Res.

[42] Li G, Yan X (2023). Long non-coding RNA GAS5 promotes cisplatin-chemosensitivity of osteosarcoma cells via microRNA-26b-5p/TP53INP1 axis. J Orthop Surg Res.

[43] Sethy C, Kundu CN (2021). 5-fluorouracil (5-FU) resistance and the new strategy to enhance the sensitivity against cancer: implication of DNA repair inhibition. Biomed Pharmacother.

[44] Blondy S, David V, Verdier M, Mathonnet M, Perraud A, Christou N (2020). 5-fluorouracil resistance mechanisms in colorectal cancer: from classical pathways to promising processes. Cancer Sci.

[45] Zhang Q, Liu RX, Chan KW, Hu J, Zhang J, Wei L (2019). Exosomal transfer of p-STAT3 promotes acquired 5-FU resistance in colorectal cancer cells. J Exp Clin Cancer Res.

[46] He WT, Zhu YH, Zhang T, Abulimiti P, Zeng FY, Zhang LP (2019). Curcumin reverses 5-fluorouracil resistance by promoting human colon cancer HCT-8/5-FU cell apoptosis and down-regulating heat shock protein 27 and p-glycoprotein. Chin J Integr Med.

[47] Hu BY, Gu YH, Cao CJ, Wang J, Han DD, Tang YC (2016). Reversal effect and mechanism of Ginkgo biloba exocarp extracts in multidrug resistance of mice S180 tumor cells. Exp Ther Med.

[48] Wang PP, Luan JJ, Xu WK, Wang L, Xu DJ, Yang CY (2017). Astragaloside IV downregulates the expression of MDR1 in Bel-7402/Fu human hepatic cancer cells by inhibiting the JNK/c-Jun/AP-1 signaling pathway. Mol Med Rep.

[49] Li P, Zhang X, Wang L, Du L, Yang Y, Liu T (2020). lncRNA HOTAIR contributes to 5FU resistance through suppressing miR-218 and activating NF-κB/TS signaling in colorectal cancer. Mol Ther Nucleic Acids.

[50] Gao S, Ren S, Liu Y, Yan H, Chen X (2021). Targeting EGFR sensitizes 5-Fu-resistant colon cancer cells through modification of the lncRNA-FGD5-AS1-miR-330-3p-Hexokinase 2 axis. Mol Ther Oncol.

[51] Tay Y, Rinn J, Pandolfi PP (2014). The multilayered complexity of ceRNA crosstalk and competition. Nature.

[52] Qi X, Zhang DH, Wu N, Xiao JH, Wang X, Ma W (2015). CeRNA in cancer: possible functions and clinical implications. J Med Genet.

[53] Zhang J, Jin M, Chen X, Zhang R, Huang Y, Liu H (2018). Loss of PPM1F expression predicts tumour recurrence and is negatively regulated by miR-590-3p in gastric cancer. Cell Prolif.

[54] You LN, Tai QW, Xu L, Hao Y, Guo WJ, Zhang Q (2021). Exosomal LINC00161 promotes angiogenesis and metastasis via regulating miR-590-3p/ROCK axis in hepatocellular carcinoma. Cancer Gene Ther.

[55] Song Q, Chen Q, Wang Q, Yang L, Lv D, Jin G (2018). ATF-3/miR-590/GOLPH3 signaling pathway regulates proliferation of breast cancer. BMC Cancer.

[56] Scott KL, Chin L (2010). Signaling from the Golgi: mechanisms and models for Golgi phosphoprotein 3-mediated oncogenesis. Clin Cancer Res.

[57] Hua X, Xiao Y, Pan W, Li M, Huang X, Liao Z (2016). miR-186 inhibits cell proliferation of prostate cancer by targeting GOLPH3. Am J Cancer Res.

[58] Wang JH, Chen XT, Wen ZS, Zheng M, Deng JM, Wang MZ (2012). High expression of GOLPH3 in esophageal squamous cell carcinoma correlates with poor prognosis. PLoS ONE.

[59] Ouyang J, Song F, Li H, Yang R, Huang H (2020). miR-126 targeting GOLPH3 inhibits the epithelial-mesenchymal transition of gastric cancer BGC-823 cells and reduces cell invasion. Eur J Histochem.

[60] Sun J, Yang X, Zhang R, Liu S, Gan X, Xi X (2017). GOLPH3 induces epithelial-mesenchymal transition via Wnt/beta-catenin signaling pathway in epithelial ovarian cancer. Cancer Med.

[61] Yu T, An Q, Cao XL, Yang H, Cui J, Li ZJ (2020). GOLPH3 inhibition reverses oxaliplatin resistance of colon cancer cells via suppression of PI3K/AKT/mTOR pathway. Life Sci.

